# A Presumed Etiology of Kawasaki Disease Based on Epidemiological Comparison With Infectious or Immune-Mediated Diseases

**DOI:** 10.3389/fped.2019.00202

**Published:** 2019-05-21

**Authors:** Jung-Woo Rhim, Hyun Mi Kang, Ji-Whan Han, Kyung-Yil Lee

**Affiliations:** ^1^Department of Pediatrics, College of Medicine, The Catholic University of Korea, Seoul, South Korea; ^2^Department of Pediatrics, Daejeon St. Mary's Hospital, The Catholic University of Korea, Daejeon, South Korea

**Keywords:** Kawasaki disease, etiology, epidemiology, acute pyelonephritis, exanthem subitum, microbiota

## Abstract

**Background:** Kawasaki disease (KD) may be associated with infection of unknown pathogen(s). For predicting of the etiology of KD, we evaluated epidemiological characteristics in KD, common infectious diseases and immune-mediated diseases in childhood.

**Methods:** We respectively, reviewed the data of patients with KD, influenza, aseptic meningitis, exanthem subitum (ES), *Mycoplasma pneumoniae* (MP) pneumonia, acute pyelonephritis (APN), Henoch-Schönlein purpura (HSP), acute poststreptococcal glomerulonephritis (APSGN), and childhood asthma. We compared and interpreted epidemiological data across the groups.

**Results:** In age distribution, KD, APN, and ES showed a similar pattern in that majority of patients were infants or young children, and other diseases showed a relatively even age-distribution which had a peak age, mainly 5–6 years, with bell-shape patterns. In annual-case pattern, there were epidemic years in aseptic meningitis and MP pneumonia, and the fluctuated annual cases were seen in other diseases. The trends of decreasing cases were seen in APSGN, HSP, and childhood asthma in recent years. In seasonal frequency, influenza or aseptic meningitis occurred in mainly winter or summer season, respectively. HSP and APSGN cases had less in summer, and KD, APN, and ES showed relatively even occurrence throughout a year without significant seasonal variations.

**Conclusions:** Our results suggest that KD agents may be associated with normal flora that are influenced by environmental changes, since pathogens of APN and ES could be regarded as normal flora that originate from the host itself or ubiquitously existing human reservoirs.

## Introduction

Kawasaki disease (KD) disease was a novel disease that appeared in East Asian countries in the order of Japan, South Korea, Taiwan, and China with a 5–10 year time-gap (1–4). After the first case report in the 1960s in Japan, KD has been reported in over 60 countries around the world (5). However, the incidence of KD is different across various populations; currently, the incidence in Western countries is lower by one tenth to one twentieth compared to East Asian countries, and has plateaued during recent decades. Whereas, the number of KD patients in East Asian countries has slowly and steadily increased after its first emergence (6). In these countries, KD occurs mostly in children between 6 months and 4 years of age, and has become a nationwide endemic disease within 2 decades after its first appearance. These findings suggest that KD spreads slowly to other regions within a nation and neighboring countries, which is contrasting to common infectious disease epidemics. KD pathogen(s) should satisfy with the epidemiological characteristics of KD.

The clinical manifestations of Kawasaki disease share similarities in some aspects of viral diseases, bacterial diseases, or infection-related immune mediated diseases such as systemic juvenile idiopathic rheumatoid arthritis or acute rheumatic fever (ARF). However, extensive studies searching for the etiologic agent(s) have turned up as failures until the present time (7). Common childhood infectious diseases and infection-related immune-mediated diseases, including KD, childhood idiopathic thrombocytopenia, and Henoch-Schönlein purpura (HSP) have low prevalence in older children and adults. Thus, these childhood diseases have been hypothesized to be associated with infections caused by unknown pathogens (8, 9). Since KD is an acute self-limiting systemic inflammation that involves multiple organs, it has been proposed that there are etiologic substances that induce systemic inflammation and target cell injuries, including coronary artery lesions (CALs), during or after the infection with KD pathogen (s) (10). Also, it has been proposed that KD pathogens may be a species of the host's normal flora based on clinical and epidemiological characteristics of KD (11). The epidemiological characteristics of a disease may aid in predicting the etiologic agent(s) of the disease. The identification of KD etiology is essential for understanding the disease, and the development of diagnostic tools and adequate treatment modalities.

In this study, to predict the etiology of KD, we evaluated and compared epidemiological characteristics between KD and common infectious diseases or immune-mediated diseases in children. After evaluating epidemiological parameters focused on age distribution, annual-case pattern and monthly-case pattern, we found that the epidemiological profiles in KD were similar to those in acute pyelonephritis (APN) or exanthema subitum (ES). We discussed the implications of these findings in KD epidemiology in Korea.

## Materials and Methods

The subjects of this study were collected from the patients (0–15 years of age) who were admitted at The Catholic University of Korea Daejeon St. Mary's Hospital from January 1987 to December 2016. We evaluated the epidemiological characteristics of patients diagnosed with *Mycoplasma pneumoniae* (MP) pneumonia, influenza, aseptic meningitis, APN, ES, acute poststreptococcal glomerulonephritis (APSGN), HSP, childhood asthma (or recurrent wheezing episode), and KD. As an exception, the subjects diagnosed with influenza in this study were selected from outpatients who were positive for influenza in rapid diagnostic testing during the winter of influenza seasons. The diagnosis or selection criteria in each disease were referenced from other publications (12–20). Although the study period and the number of patients in each disease were not identical, we reevaluated the data that were used for previously published papers or collected new data for some diseases such as ES and HSP. Age, sex, age distribution pattern, annual-case pattern and monthly-case pattern were analyzed in each disease. For mean age, <12 months of age was regarded as 0 years of age for statistical analyses, except ES. In age distribution pattern, we focused on age predilection in infancy and younger children. In annual-case pattern, we searched whether the pattern shown cyclic epidemics during the study periods. As for seasonal variation, the disease was regarded as having seasonality when the number of seasonal cases showed over 10 or 15% difference in the number of cases between the highest season and the lowest season.

### Ethics Statement

The written informed consents were obtained from the parents/caregivers of all children for the medical records to be used in this study at time of admission. The study was approved by the Institutional Review Board of The Catholic University of Korea Daejeon St. Mary's Hospital (DC18RESI0100).

## Results

A total of 7,832 patients diagnosed with 9 diseases, including KD, 5 infectious diseases, and 3 immune-mediated diseases were evaluated. The study period in the majority of diseases was over 10 years; for infectious diseases, MP pneumonia (2003–2012, *n* = 779) (12), influenza (2010–2017, *n* = 2,163) [(13, 14), unpublished observation], aseptic meningitis (1987–2003, *n* = 2,201) (15), APN (2005–2015, *n* = 320) (16) and ES (2005–2016, *n* = 429), and as for infection-related immune diseases, APSGN (1987–2013, *n* = 99) (17), HSP (1987–2015, *n* = 515) (unpublished observation) and childhood asthma (or recurrent wheezing episode) (2003–2014, *n* = 384) (18), and KD (1987–2016, *n* = 942) (19, 20) (Table 1).

**Table 1:** Demographic findings in KD and other diseases (0–15 years of age).

**Diseases**	**Study period**	**No. of subjects**	**Mean cases/y**	**M:F ratio**	**Mean age (y)**	**Peak ages (y)**
Kawasaki disease	1987–2016	942	31	1.7:1	2.2 ± 1.6	1
Mycoplasma pneumonia	2003–2012	779	78	1.1:1	5.0 ± 3.2	4
Influenza	2010–2018	2,163^*^	270	1.1:1	5.4 ± 3.8	3–5
Aseptic meningitis	1987–1998	2,201	115	2:1	6.0 ± 3.9	4–6
Acute pyelonephritis	2005–2015	320	29	1.4:1	1.5 ± 3.4	0
Exanthem subitum	2005–2016	429	36	1:1	1.0 ± 0.5	0
APSGN	1987–2014	99	4	2.3:1	8.3 ± 2.7	7–9
Henoch-Shönlein purpura	1987–2015	515	18	1.2:1	6.5 ± 3.0	5
Childhood asthma	2003–2014	384	32	1.2:1	5.6 ± 3.5	2–4

* *Outpatients; APSGN, acute poststreptoccocal glomerulonephritis*.

### Age, Sex, and Age Distribution in KD and Other Diseases

The male-to-female (M:F) ratio, mean age, and peak ages are shown in Table 1.

**Figure 1:**
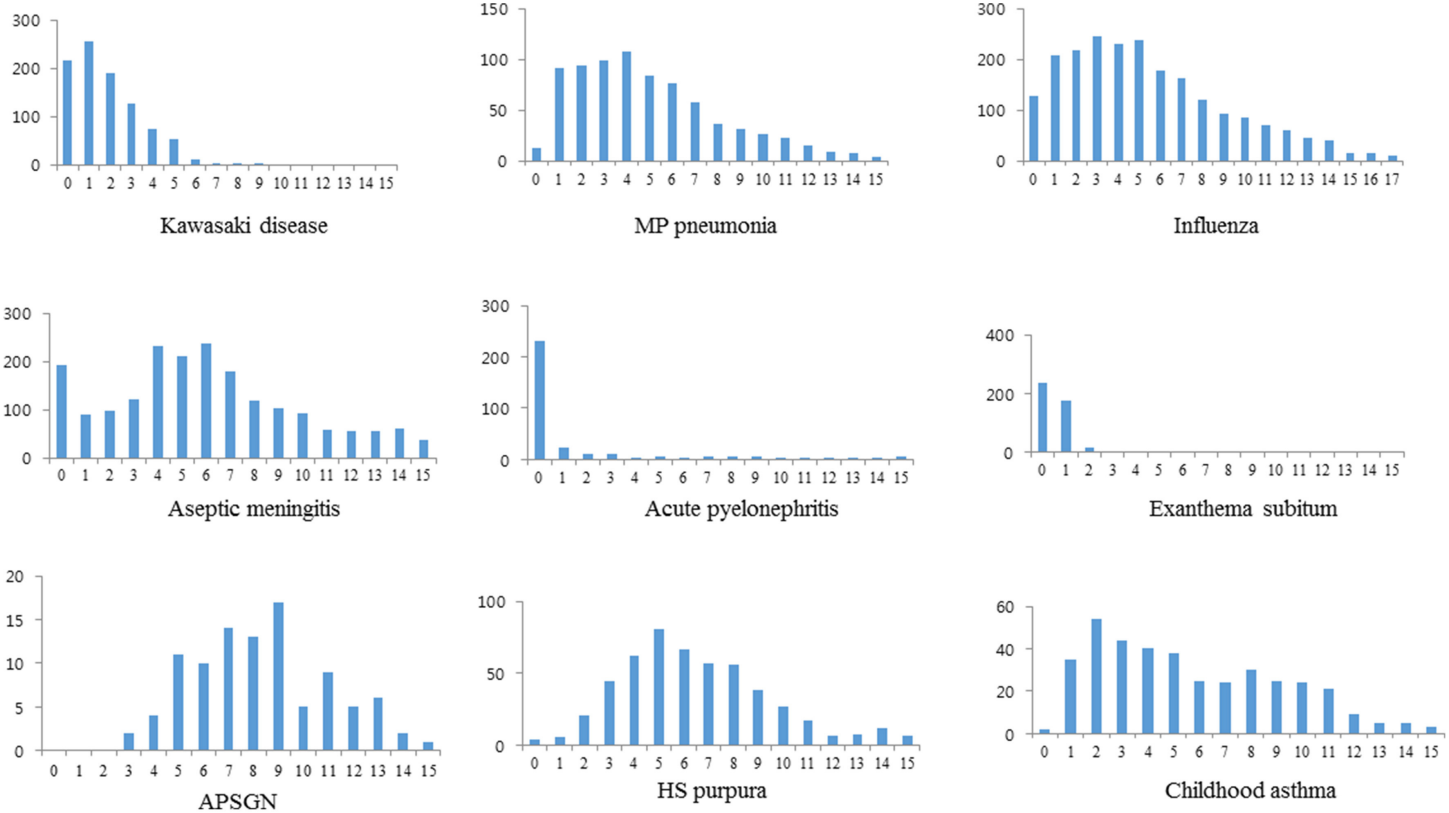
Age distribution in Kawasaki disease and other infectious or infection-related immune-mediated diseases.

Male predominance was observed in all the diseases except ES (0.95:1), but the M:F ratios were somewhat different across the diseases. The highest M:F ratio was seen in APSGN (2.3:1), and the lower M:F ratios were seen in ES, MP pneumonia and influenza (1.1:1, respectively), and KD showed 1.7:1 ratio in this series. The lowest mean age was noted in order of ES (mean 1 year of age), APN (1.5 years), and KD (2.2 years) and the higher mean age was noted in APSGN (8.3 years) and HSP (6.5 years). In age distribution, KD, APN, and ES showed a similar pattern in that the majority of patients were infants and young children (0–4 years), and after this age period the prevalence decreased dramatically. There were only a few patients >3 years of age in ES and a few patients >5 years in KD, however there was relatively an even distribution with female predominance after >2 years in APN. A bell-shape distribution pattern with a peak prevalence, mainly in the 4–6 years age range was observed in other diseases, however peak ages were slightly different across the diseases (Figure 1). There was a trend where the peak ages were changed in the epidemic diseases such as MP pneumonia and influenza over time [(12), unpublished observation].

### Annual-Case Patterns in KD and Other Diseases

**Figure 2:**
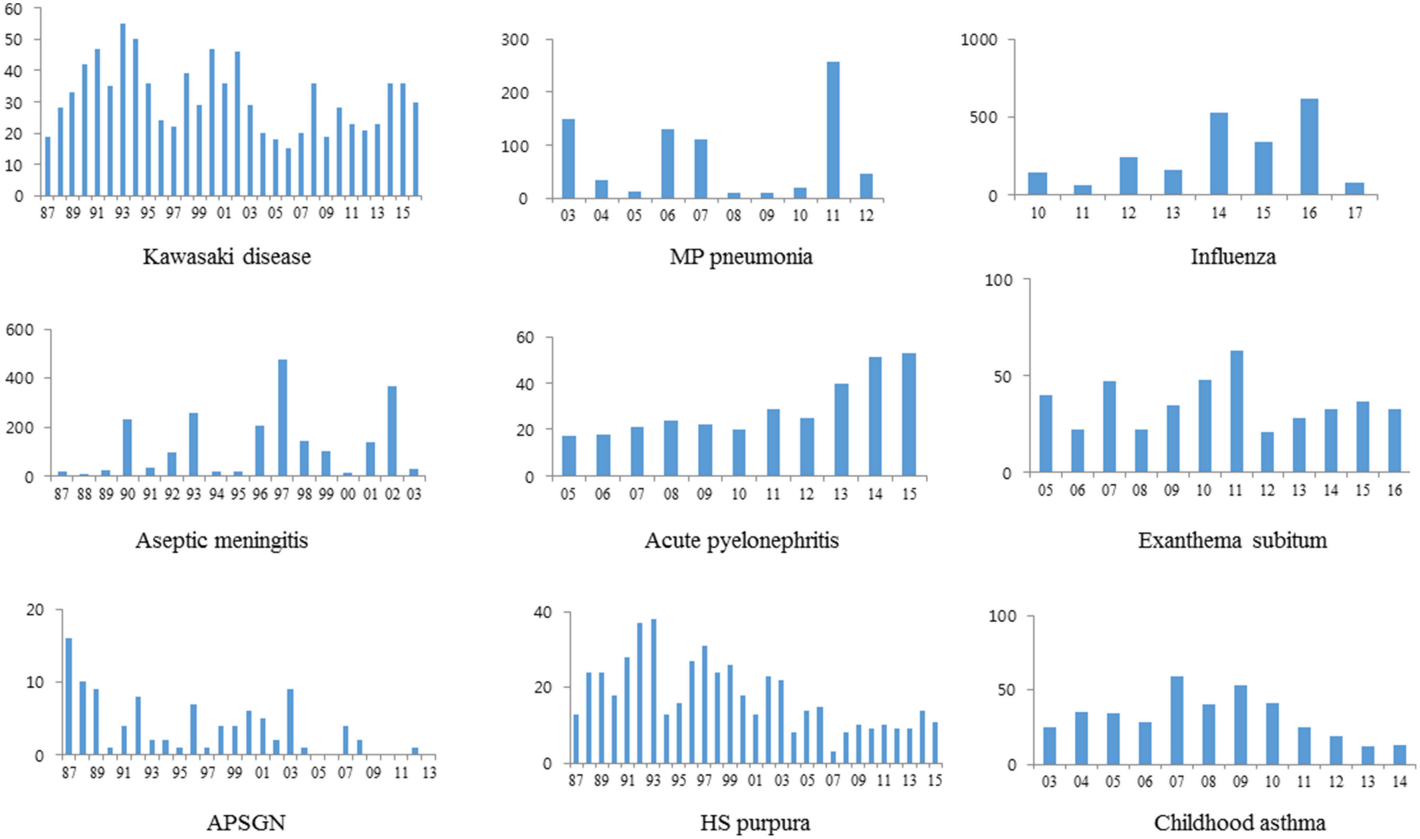
Annual cases in KD and other diseases.

Cyclic epidemics caused by possibly new strains of pathogens, such as macrolide-resistant strains were noted in MP pneumonia and in aseptic meningitis caused by mainly enteroviruses; all had similar patterns with nationwide epidemics or outbreaks in Korea (12, 15). Influenza occurred every winter and early spring, but the number of cases and peak epidemic months were different in 2009 pandemic and seasonal influenza, across seasonal influenza after 2009 pandemic [(14), unpublished observation]. Relatively an even, but fluctuating annual cases were noted in KD, APN, ES, HSP, and childhood asthma, except APSGN (Figure 2). The mean annual cases in each disease are shown Table 1, and the results may be helpful to estimate the incidence of each disease in our city, since nationwide KD epidemiological studies in Korea have been performed every 3 years (21, 22). There was a trend of decreasing number of cases in the recent years or decades in APSGN, HSP and childhood asthma, whereas an increasing trend was noted in APN, compared to the past years or decades (Figure 2).

### Monthly or Seasonal-Case Pattern in KD and Other Diseases

**Figure 3:**
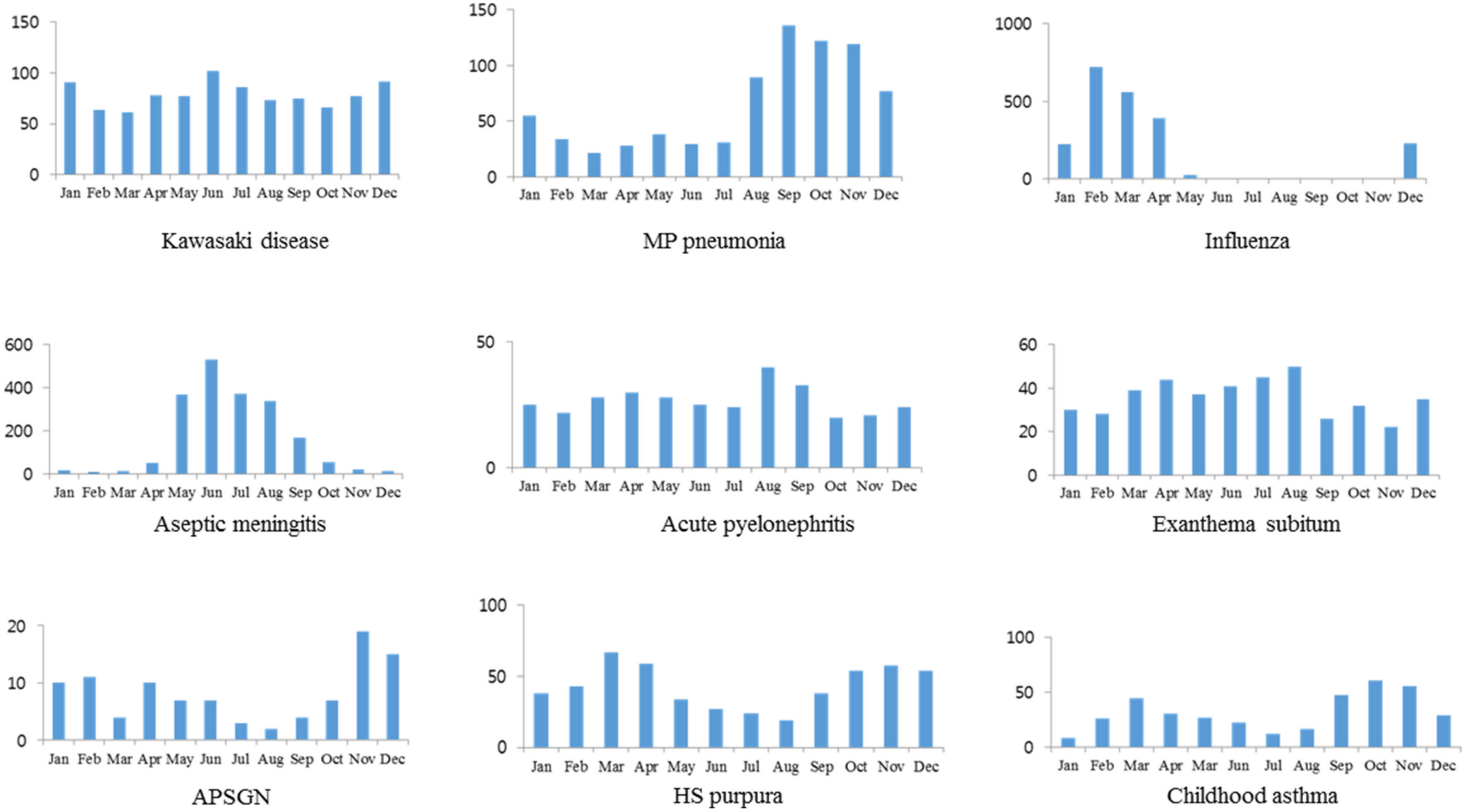
Monthly or seasonal distribution of cases in KD and other diseases.

A higher number of cases were seen in the fall and winter in MP pneumonia. Aseptic meningitis was noted mainly during the summer whereas influenza mainly during winter and early spring seasons. There was a trend showing a lower number of cases in the summer in HSP, and a higher number of cases in the winter for APSGN. A higher number of cases were seen during the fall and spring for childhood asthma. Although KD affected more patients during summer than any other season in this series, KD had no difference in seasonal variation according to the definition of the seasonality. Also, no seasonality was seen in APN and ES (Table 2 and Figure 3).

**Table 2:** Seasonality in KD and other diseases.

**Diseases**	**Spring (%)**	**Summer (%)**	**Fall (%)**	**Winter (%)**	**Seasonality**
Kawasaki disease	22.9	27.7	23.1	26.2	No seasonality
Mycoplasma pneumonia	11.2	19.1	48.4	21.3	Fall predominance
Influenza	45.3	0.2	0.1	54.4	Spring, winter
Aseptic meningitis	22.2	63.5	12.3	1.9	Summer predominance
Acute pyelonephritis	26.9	27.8	23.1	22.2	No seasonality
Exanthem subitum	28.4	31.7	18.6	21.8	No seasonality
APSGN	21.2	12.1	30.3	36.4	Winter, fall
Henoch-Shönlein purpura	31.1	13.6	29.1	26.2	Low in summer
Childhood asthma	26.8	13.5	43	16.7	Fall, Spring

*APSGN, acute poststreptococcal glomerulonephritis*.

## Discussion

It is hypothesized that KD may have an etiologic agent(s), although many studies searching for the etiology of KD have failed. Etiologic agents in infectious diseases originate from an external source and invade into the host. However, at present time, they could be classified as exogenous pathogens and the endogenous pathogens; the former is generally accepted as agents that are newly introduced to humans from other animal species or other places, whereas the latter originates from the host in the forms of normal flora (or commensals) or those becoming a normal flora from initially being an exogenous pathogen. For examples, measles in immune-innocent populations in the past and acquired immunodeficiency syndrome (AIDS) were caused by new external pathogens. On the other hand, the pathogens causing APN or acute otitis media, such as *E. coli* or *Streptococcus pneumoniae*, could be categorized as endogenous pathogens. It is well-known that a newly introduced disease by an exogenous pathogen has affected all aged persons in a population and spread to other populations in relatively a short-time period of time after its first emergence. Over time, infected persons and groups have immunity to the pathogens, and young children and infants would remain susceptible, especially during cyclic epidemic viral infections. Accordingly, epidemiologic characteristics of infectious diseases or infection-related diseases in the populations at a given time may differ.

In the present study, we found that epidemiological characteristics in KD were most similar to those in APN or ES among the 8 evaluated diseases. In age distribution, these 3 diseases had an age predilection in infancy and young children; 79.7% of patients in APN, 95.8% in ES, and 50.1% in KD were 0–1 years of age, and 87.5% of patients in APN, near 100% in ES, and 91.7% in KD were 0–4 years of age. In the view of epidemiological and clinical aspects, an infected infant with pathogens from these diseases are difficult to disperse the pathogens to other infants. Other diseases showed a relatively even age-distribution throughout childhood, though peak ages were slightly different across the diseases. This suggests that younger children may have less chance on exposure to the pathogens and older children and young adult groups may have immunity or tolerability to the pathogens causing the diseases.

In the annual-case pattern, cyclic epidemics were noted in MP pneumonia and aseptic meningitis, and marked fluctuated cases with a different peak week were noted in influenza. The annual-case pattern in KD was relatively even, but fluctuations in annual cases were noted as well as in APSGN, HSP and childhood asthma, and APN and ES in infectious diseases. APN may be the most common systemic bacterial infection in early childhood in developed countries (23). A majority of uropathogens causing APN in infants are *E. coli* or other uropathogens such as *Klebsiella* spp., *Proteus* spp., and *Enterococcus* spp., and they may originate from the intestinal commensals in the host. It is possible that after colonization of a strain of *E. coli* or other uropathoegns transferred from other persons, the pathogens invade into the host on occasional events and elicit immune reactions (24). Although KD and APN affect mainly infants and young children, older children and adults are also affected (25). Moreover, recurrent cases are not uncommon in the both diseases. These findings suggest that immature immune function in early childhood may be associated with both diseases, and pathogens may be multiple in KD (11). ES is an acute systemic viral infection, and is characterized by sudden appearances of generalized maculopapular or morbilliform rashes just after defervescence. The etiologic agent is a species belonging to the human Herpes virus group, herpes virus type 6 or rarely type 7. Herpes viruses have a characteristic of latent infections after initial infection. ES may have no differences in age predilection, possibly sero-prevalence rate, and clinical manifestations across populations around the world and have occurred throughout every year with no seasonal variations (26, 27). Herpes virus groups become latent in the host after the initial infection as shown in herpes zoster, herpes labialis, and reactivation of cytomegalovirus and Epstein-Barr virus (EBV) in depressed immune state of the host (28). It was reported that EBV infection or herpes virus 6 infection was related to certain clinical manifestations in KD such as otorrhea or BCG inoculation site inflammation (29, 30). It is a reasonable presumption that etiologic viruses in ES may have been introduced to humans a long-time ago, and may be coexisting within healthy human reservoirs including family members of infected infants with ES, and can be activated at any time. On occasion, the latent viruses in healthy carriers who may have a transient immune disturbance, can be reactivated and be spread to infants who have no immunity to the viruses.

In the present study, monthly case or seasonal case patterns in KD were also the most similar to those in APN or ES. Similar to this study, Nagao et al. (31) reported that the super-annual periodicity of KD was the most similar to ES among seven childhood infectious diseases including ES, herpangina, hand-foot-mouth disease, chicken pox, pharyngoconjunctival fever, erythema infectiosum, and GAS infection. They suggested that the KD pathogen is transmitted through close contact and persists asymptomatically in most hosts, likely ES (31), and this suggestion is accordance with our presumed characteristics of KD pathogens. Although the rate of KD has slowly increased with slight seasonal predominance in nationwide studies in Japan and Korea (22, 32), KD has occurred with annual fluctuations with seasonal variation each year in a given location as shown in this study. These data suggest that KD appears as local outbreaks rather than as a simultaneous nationwide epidemic as shown in viral or MP infections in Korea.

We have proposed that KD pathogens may be a species of normal flora of the host (10, 11). The disturbance of microbiota of the host, i.e., dysbiosis, has been reported in a variety of diseases, including obesity, autism spectrum disorders, allergic diseases, cancers, and autoimmune diseases such as inflammatory bowel disease, JIA and KD (33–38). It has been known that microbiota differ across ethnic groups along with different cultural environments such as diets, antibiotic use, and possibly genetic factors (39–41). Kinumaki et al. reported that *Streptococcus* spp. in intestinal microbiota markedly increased during the acute phase of KD, and suggested that KD-related streptococci may be involved in the pathogenesis of KD (38). Recently, Esposito et al. reviewed the possible role of the microbiota-host partnership on etiology and pathogenesis of KD (42). Because normal flora spread via colonization in individuals in populations, a new disease that is associated with normal flora may take a long period of time during which to spread, and would show different incidences across populations at given times as shown in KD epidemiology in Asian countries. Since pathogens exist in healthy human reservoirs as normal flora, the diseases may occur mainly in immunologically or genetically susceptible groups such as young children group that have developing immune system and microbiota (43, 44). Also, the epidemiology in these diseases can change over time along with an increasing number of people who obtain pathogens as normal flora in the populations, since normal flora that have adapted to the host may be less virulent compared to initial external pathogens. For example, scarlet fever caused by Group A beta-hemolytic streptococcus (GAS) was a severe disease in the past, with two notorious complications: acute rheumatic fever (ARF) and APSGN. Now, scarlet fever, ARF and APSGN have become rare diseases with a milder clinical phenotype in the developed countries (17, 45). However, pharyngitis caused by GAS without complications is not uncommon, and a relatively high proportion of GAS carriers among healthy children have been reported in the developed countries (46, 47). In addition, GAS strains have been susceptible to penicillin throughout the antibiotic era, with few genetic variations (48). Given that the evolutional purpose of external pathogens may be to become a species of normal flora in the host, these findings suggest that GAS strains may be changing to be part of normal flora in humans, though ARF still occurs in small populations (49). Thus, it is natural that initially severe diseases take on milder phenotypes over time, as shown in scarlet fever, pandemic influenza, and AIDS (13, 45, 50). It was also reported that recently diagnosed KD patients showed a less severe clinical phenotype, manifesting a higher incidence of incomplete KD and a lower incidence of CALs and less activated laboratory values such as C-reactive protein and albumin compared to patients who developed the disease in earlier periods (51).

Pathogenesis of KD and APN as well as other infectious and immune-mediated diseases remains to be further studied. APN has a focus in renal cortical parenchyma, where replicated uropathogens, byproducts from APN agents such as toxins and pathogen-associated molecular patterns (PAMPs), substances from injured host cells, and those from activated immune cells are produced. When these diverse substances spread from the focus into systemic circulation, clinical manifestations such as fever, tissue cell injury and/or bacteremia begin to occur, and the host immune system may respond to the substances. The majority of patients infected with APN pathogens may be asymptomatic and their disease are self-limited if systemic spread did not occur and the substances in the focus were controlled as a localized inflammation (24). On the other hand, ARF or APSGN are classic representatives of infection-related immune-mediated disease. In ARF, after 1–4 weeks after initial GAS infection when symptoms and signs of the initial bacterial infection (pharyngitis) subside, acute onset of fever and other symptoms, such as carditis, arthritis, and rarely skin rash (erythema marginatum), develop (49). The majority of patients with ARF or APSGN show a self-limited clinical course, although severely affected patients have long-term complications such as severe rheumatic heart disease or chronic renal failure, similar to giant coronary artery aneurysms in KD. Only a small proportion of patients with GAS infection are affected with ARF or APSGN. Some patients with ARF or APSGN may have preceding asymptomatic GAS infections, and other group streptococci, other bacteria or viruses are associated with postinfectious glomerulonephritis or heart tissue inflammation, including myocarditis and possibly heart valve diseases, without evidence of GAS infection (52, 53). It is believed that there are etiologic substances that induce various clinical manifestations in KD as well as in ARF and APSGN after initial infection (10). These substances include not only those originating from the pathogens but also those from the injured host cells, including damage-associated molecular patterns (DAMPs) and those from immune cells activated by infectious insults. It have been proposed that these substances bind to specific target cells of organs such as heart and joints in ARF, kidneys in APSGN, or coronary arteries or other organs in KD. The host immune system may control these substances based on their size and biochemical properties (the protein-homeostasis-system hypothesis) (10, 54, 55).

Based on the data from epidemiological and clinical studies of KD, the pathogenesis of KD could be explained as follows. Along with economic growth and westernization in East Asian countries in order of Japan, South Korea, Taiwan, and China, a species in normal flora (microbiota) in people may be affected by environmental changes such as improved hygiene or increased consumption of westernized foods including red meats (10, 11). These species (KD agents) slowly spread via colonization as normal flora to other people, including the parents and guardians of KD patients, and are eventually colonized in infants and young children predisposed to KD. On occasion, colonized KD agents invade the host via unknown events; if they are intestinal commensals, a mechanism similar to APN (via hematogeneous route in young infants) may be activated (24). Invading KD pathogens make a focus (replication site) elsewhere within the host, possibly near the upper respiratory or intestinal tracts (portal of entry) or in the target organ via systemic or local circulation. The majority of patients infected by the KD agents may be asymptomatic and have self-limited disease. However, during or after recovery from this infection, similar to ARF or APSGN, KD develops when the substances from a focus spread abruptly into systemic circulation (10). There are various incubation periods in ARF and APSGN after initial GAS infection, and no intact pathogens or structural components of the pathogen have been detected in the pathologic lesions in ARF, APSGN, KD, and other infection-related immune-mediated diseases. Furthermore, GAS strains can reside in intracellular compartments in host cells such as tonsillar epithelial cells and macrophages (56, 57), and small extracellular bacteria such as mycoplasma species can proliferate within cells and spread to other organ cells (58). Therefore, it is possible that the etiologic substances may originate from injured host cells, including a kind of DAMPs, or from immune cells exposed to infectious insults, than from various pathogens. Immune/repair system of the host, including immunoglobulins (IgG, IgM, and IgM) and platelets, begin to control the substances and take part in tissue cell repair (59). Thus, the prognoses of KD and other immune-mediated diseases depend on the ability of the host immune system to control the diverse substances from target cells injured by initial insults. Early immune modulators (corticosteroids or intravenous immunoglobulin) may act on hyperactive immune reactions performed by the non-specific adaptive immune cells (T cells and B cells) in the early stage of each disease (10, 54, 60). However, despite early treatment, a few patients with KD experience severe or giant aneurysms, and these patients may have insufficient immune function to control the substances originating from injured target cells (61). Young children with immature immune function may be prone to invasion of KD pathogens as occurs in APN in infants, and the probability of invasion of KD agents in young children may be similar across different populations (20). Since microbiota may differ across populations, marked differences in racial incidence in KD or other immune-mediated diseases such as HSP, inflammatory bowel disease, and Bechet disease may be associated with the colonization state of the different etiologic agents in the populations (20).

This retrospective study has some limitations. Epidemiological data observed in a single hospital may not match the nationwide data. However, we found that outbreaks of infectious diseases such as MP pneumonia, influenza, and aseptic meningitis in our city had occurred concurrently with nationwide epidemics. Demographic and some epidemiologic characteristics of KD, APN, APSGN, HSP, childhood asthma, and possibly ES were similar to the data in published papers in Korea (data not shown).

In conclusion, epidemiological characteristics of KD were similar to APN or ES in age distribution, annual case pattern, and seasonal variations. The pathogens of APN and ES could be regarded as one species of normal flora, since they may have originated from the host itself or ubiquitously existing human reservoirs. KD may also be associated with a species in the normal flora that may be influenced by environmental changes.

## Ethics Statement

The written informed consents were obtained from the parents/caregivers of all children for the medical records to be used in this study at time of admission. The study was approved by the Institutional Review Board of The Catholic University of Korea Daejeon St. Mary's Hospital (DC18RESI0100).

## Author Contributions

K-YL designed the study, collected data, contributed to interpretation of results, and drafted the manuscript. J-WR contributed to data collection and drafted the initial manuscript. HK and J-WH contributed to data collection and revised the manuscript. All authors have read and approved submission of the final version of the manuscript.

### Conflict of Interest Statement

The authors declare that the research was conducted in the absence of any commercial or financial relationships that could be construed as a potential conflict of interest.
